# Altered tryptophan and alanine transport in fibroblasts from boys with attention-deficit/hyperactivity disorder (ADHD): an in vitro study

**DOI:** 10.1186/1744-9081-7-40

**Published:** 2011-09-24

**Authors:** Jessica Johansson, Magnus Landgren, Elisabeth Fernell, Ravi Vumma, Arne Åhlin, Lars Bjerkenstedt, Nikolaos Venizelos

**Affiliations:** 1 Department of Clinical Medicine, School of Health and Medical Sciences, Örebro University, 701 82 Örebro, Sweden; 2 Department of Pediatrics, Skaraborg Hospital, Unit of Neurodevelopmental Disorders, 542 24 Mariestad, Sweden; 3 Research and Development Centre, Skaraborg Hospital, 54185 Skövde, Sweden; 4 The Gillberg Neuoropsychiatry Center, Sahlgrenska Academy, Gothenburg, Sweden; 5 Strömstad Academy, SE-45280 Strömstad, Sweden

## Abstract

**Background:**

The catecholaminergic and serotonergic neurotransmitter systems are implicated in the pathophysiology of attention-deficit/hyperactivity disorder (ADHD). The amino acid tyrosine is the precursor for synthesis of the catecholamines dopamine and norepinephrine, while tryptophan is the precursor of serotonin. A disturbed transport of tyrosine, as well as other amino acids, has been found in a number of other psychiatric disorders, such as schizophrenia, bipolar disorder and autism, when using the fibroblast cell model. Hence, the aim of this study was to explore whether children with ADHD may have disturbed amino acid transport.

**Methods:**

Fibroblast cells were cultured from skin biopsies obtained from 14 boys diagnosed with ADHD and from 13 matching boys without a diagnosis of a developmental disorder. Transport of the amino acids tyrosine, tryptophan and alanine across the cell membrane was measured by the cluster tray method. The kinetic parameters, maximal transport capacity (*V_max_*) and affinity constant (*K_m_*) were determined. Any difference between the two groups was analyzed by Student's unpaired *t*-test or the Mann Whitney U test.

**Results:**

The ADHD group had significantly decreased *V_max _*(p = 0.039) and *K_m _*(increased affinity) (p = 0.010) of tryptophan transport in comparison to controls. They also had a significantly higher *V_max_*of alanine transport (p = 0.031), but the *Km *of alanine transport did not differ significantly. There were no significant differences in any of the kinetic parameters regarding tyrosine transport in fibroblasts for the ADHD group.

**Conclusions:**

Tryptophan uses the same transport systems in both fibroblasts and at the blood brain barrier (BBB). Hence, a decreased transport capacity of tryptophan implies that less tryptophan is being transported across the BBB in the ADHD group. This could lead to deficient serotonin access in the brain that might cause disturbances in both the serotonergic and the catecholaminergic neurotransmitter systems, since these systems are highly interconnected. The physiological importance of an elevated transport capacity of alanine to the brain is not known to date.

## Background

Attention-deficit/hyperactivity disorder (ADHD) is a neurodevelopmental disorder with a prevalence in children of about 5-7% worldwide [1,2]. It is clinically characterized by a persistent pattern of inattention and/or hyperactivity-impulsivity that affect cognitive, behavioural, emotional and social functioning and symptoms may also persist into adulthood [1,3,4].

As for many other neurodevelopmental disorders no single etiology of ADHD has been provided, but a number of underlying theories exist. The disorder is highly heritable, based on family, twin and adoption studies [5], but the genetic architecture of ADHD is suspected to be complex. However, a number of candidate genes have been identified and among these are several genes associated with the catecholaminergic system [6]. Also, a recent study shows that children with ADHD have an increased rate of large copy number variants (CNVs) especially at chromosome 16 [7], which further support ADHD as a genetic disorder.

Many neuro-imaging studies have identified abnormalities of brain structure and function in ADHD and in particular, a dysfunction of the fronto-subcortical pathways in the brain has been implicated [8]. These pathways control attention and motor behaviour, and it is known that the catecholamines; dopamine and norepinephrine, are vital for their function [8]. Moreover, stimulant agents, such as methylphenidate, amphetamine and atomoxetine, which currently are the most common drugs used for treatment of ADHD, act primarily on the catecholaminergic system. Therefore, the neurotransmitters dopamine and norepinephrine have been implicated in the pathophysiology of ADHD. However, an involvement of serotonin and the serotonergic system in the pathophysiology of ADHD have also regained the researchers' interest during recent years, since there is an interaction between the dopaminergic and serotonergic neurotransmitter systems. It is suggested that serotonin can modulate the activity of dopamine and an alteration in the serotonergic neurotransmission can alter dopamine-mediated behaviour [9,10]. Still, the relevance of serotonin in ADHD needs to be further explored.

The rate of synthesis of the neurotransmitters dopamine, norepinephrine and serotonin in the central nervous system (CNS) is partly dependent on the brain's availability of precursor amino acids [11]. The amino acid tyrosine is the precursor for the synthesis of dopamine and norepinephrine, while tryptophan is the precursor for the synthesis of serotonin and both amino acids are essential for the brain. Amino acids are polar molecules and are therefore actively transported across cell membranes, like the endothelial cells that constitute a part of the blood brain barrier (BBB), by different amino acid transport systems [12-14]. A competition between amino acids using the same transporters exists [13,15]. A number of different amino acid transport systems, with different amino acid selectivity, have been identified to date, such as system L, system A, and system ASC [12].

In order to study amino acid transport properties, in various psychiatric disorders, fibroblast cells are being used as a human experimental model. This model is suggested to be relevant since fibroblasts have a similar expression of amino acid transporters as human brain microvascular endothelial cells (hBME) and express several neuronal specific receptors and enzymes [15-18]. Recent studies have also shown that fibroblast cells can be converted into functional neurons [19]. Moreover, they are easily obtained from both patients and controls, and in comparison to other cell types they can be grown in larger amounts, are stable for many generations and are not affected by prior medication status of the patient [16].

The transport of both tyrosine and tryptophan has recently been characterized in fibroblast cells. Tyrosine was found mainly to be transported by system L, with LAT1 as the main transporter [15], in accordance with the transport of tyrosine through human brain microvascular endothelial cells [18,20,21]. Tyrosine is also, to a less extent, transported through system A, by its isoform ATA2 [12]. System A is known to transport short-chain amino acids, such as alanine, but in a study by Vumma et al in 2008 [15] it was demonstrated that approximately 50% of alanine was transported through the LAT1 isoform of system L, which denotes a competition between alanine and tyrosine to get transported. The transport of tryptophan in fibroblast cells is mediated by multiple transporters that are active at different substrate concentrations [22]. However, at physiological tryptophan plasma concentrations the transport is mainly through the LAT1 isoform of system L [22].

When using the fibroblast cell model, previous findings by our group [23-29] and others [30] have shown a disturbed membrane transport of tyrosine in a number of psychiatric disorders, such as schizophrenia, autism and bipolar disorder, Moreover, in a previous study by our group, we found that children with autism had an increased transport capacity of alanine across cell membranes [24]. An elevated transport of alanine across the BBB might influence the transport of other amino acids that are of vital importance for normal brain activity, since amino acids using the same transporter compete for transport [13,15].

The aim of this study was to investigate whether children with ADHD have changes in the transport of tyrosine and/or tryptophan, since these amino acids are the precursors for the neurotransmitters implicated in the pathophysiology of ADHD. Moreover, as there is a competition between tyrosine and alanine we studied the transport of alanine in children with ADHD.

## Materials and Methods

### Children with ADHD

The study included fibroblast cell lines from 14 boys with ADHD of the combined type (inattention combined with hyperactivity and impulsivity), according to the DSM-IV criteria [31]. They were between 6-12 years old (mean 10 years) at the time of the study. All were patients at the Unit of Neurodevelopmental Disorders, Department of Pediatrics, Skaraborg Hospital, Mariestad which is a primary pediatric referral center for pharmacological treatment of children and adolescents with ADHD. The patients had been diagnosed by an experienced team within the unit with support from standardized ADHD-rating scales used by clinicians, teachers and parents as well as with a general pediatric and psychological/cognitive work up. First grade relatives with ADHD or disruptive disorder, in addition to an uneventful pregnancy and perinatal period, were considered as an indication of heredity for ADHD. There were some true co-morbidities i.e., two boys had migraine and two other boys had asthma and still three others were overweight. Symptoms for motor coordination disorder, oppositional defiant disorder, conduct disorder, intellectual disability and autism spectrum disorder were checked for but not diagnosed at the time for the study. Both intellectual disability and autism spectrum disorder were exclusion criterias. All children were pharmacologically treated, twelve with methylphenidate and two with atomoxetine.

### Comparison group

The comparison group consisted of fibroblast cell lines from 13 boys matched for age and without a diagnosis of a developmental disorder, between 7 and 13 years old (mean 10 years). Five of the 13 cell lines were used as controls in a previous study and were obtained from a Biobank [24]. Eight skin biopsies were taken in connection with ear-nose and throat surgery (such as insertion of transmyringeal ventilation tubes).

### Collection of biopsies

A 2 mm^2 ^skin punch biopsy was taken after anaesthetizing the mid-forearm under aseptic conditions as described previously [24]. The tissue was immediately placed in tubes containing complete culture medium and transported to the laboratory. From the biopsy was fibroblast cell lines cultured and stored in a Biobank (-196°C) until used for the experiments.

### Materials

All growth media, antibiotics and fetal bovine serum (FBS) were obtained from Gibco Invitrogen cell culture (Sweden). 

Tissue culture flasks and multi-well plates were from Costar Europe Ltd, Costar NY. ^14^C (U)-L-tyrosine with specific activity 486 mCi/mmol, ^3^H(5)-L-tryptophan with specific activity 30 Ci/mmol and ^14^C(U)-L- Alanine with specific activity 110 Ci/mmol were obtained from Larodan Fine Chemicals AB (Malmö, Sweden). D-Glucose was obtained from Ambresco (Ohio, USA) and phosphate buffered saline (PBS) was from the National Veterinary Institute (SVA) (Uppsala, Sweden). All other chemicals and amino acids were purchased from Sigma-Aldrich Sweden AB (Sweden). Scintillation cocktail (Optiphase, Hisafe 3) and liquid scintillation counter (Winspectral 1414) were from PerkinElmer Life Sciences (USA). Scintillation vials were purchased from Sarstedt AB (Sweden). Micro-well plates used for protein determination were purchased from Nunc (Roskilde, Denmark) and readings were done using Multiscan MS from Labsystems (Helsinki, Finland). All amino acid solutions were made in PBS and the pH was maintained between 7.35 and 7.40.

### Cell culturing

Fibroblast cells were cultured in plastic tissue culture flasks containing minimal essential medium (MEM) supplemented with 10% FBS, L-glutamine (2 mM/L), penicillin (100 mg/ml), streptomycin (100 mg/ml) and Amino-Max™. Cells were maintained in a humidified atmosphere of 5% CO^2 ^in air at 37°C. Before the measurement of amino acid transport, cells were harvested when confluent and seeded in 2 cm^2^-multi-well plates and grown to confluence for approximately 5 days. Cell lines between 4^th ^and 14^th ^passages (number of splitting) were used in the experiments.

### Transport assay of amino acids

Amino acid transport was measured using the cluster tray method for rapid measurement of amino acid flux in adherent fibroblast cells [25,32,33]. Fibroblasts grown in multi-well plates were washed twice with PBS and incubated with PBS containing 1% D-glucose for 1 hour at 37°C, to deplete the endogenous amino acid pools. After removal of the pre-incubation medium, the cells were incubated for 60 seconds at 37°C with a constant amount of ^14^C(U)- L-tyrosine or ^3^H(5)-L-tryptophan or ^14^C(U)-L-alanine and 12 different concentrations (varying between 0.004 and 1.5 mmol/L for tyrosine, 0.005 to 0.5 mmol/L for tryptophan and 0.02 to 6 mmol/L for alanine) of unlabelled amino acids. Amino acid transport was terminated by rapidly washing the cells twice with ice-cold PBS. The cells were then lysed in 0.2 ml of 0.5 mol/L sodium hydroxide (NaOH) for approximately 30 minutes. The radioactivity of the cell lysate was measured by liquid scintillation counting from a mixture of cell lysate and scintillation cocktail. The total amino acid uptake was correlated to the total amount of protein in each well, determined by the Bradford method [34], using bovine serum albumin as a standard.

### Calculations

The amino acid kinetic parameters *V_max_*and *K_m_*were calculated from the obtained uptake values, corrected for diffusion constant (*K_d_*), by using the Lineweaver-Burke plot equation [1/V_0 _= (*K_m_*/*V_max_*[S] + (1/*V_max_*)] as described previously [25]. *V*_0 _is the initial transport capacity, [S] is the substrate concentration, *V*_max _is the maximal transport capacity (nmol/min/mg protein) and *K*_m_, is the affinity constant (the concentration at half-saturation, μmol/L). Each experiment was performed in duplicate at the same time point for all amino acid transport assays and a mean value was taken for kinetic analysis.

### Statistics

All kinetic parameters are presented by descriptive statistics (mean with standard deviations or median with range). Assumptions about parametric methods were fulfilled for *K_m_*of tyrosine, tryptophan and alanine transport, but not for *V_max_*of tyrosine, tryptophan and alanine transport (determined using Shapiro-Wilk *W *test).

Significance of the difference in *K_m_*for tyrosine, tryptophan and alanine transport between children with ADHD and the comparison group was analysed using the Student's unpaired *t*-test. Significance of the difference in *V_max _*for tyrosine, tryptophan and alanine transport between children with ADHD and the comparison group was analysed using the Mann Whitney U test.

For all statistical analyses a significant level of 5% (two-tailed) was accepted. All statistical analyses were performed using PASW statistics version 18.0 for Windows.

The study was approved by the Regional Ethics Committee in Gothenburg, Sweden (Dnr: 218-07). A written and signed consent was obtained from the parent/s and their child before performing the studies.

## Results

Maximal transport capacity (*V_max_*) and mean affinity of binding site (*K_m_*) of tyrosine, tryptophan and alanine transport in children with ADHD and in controls are presented in Table 1.

**Table 1:** Kinetic parameters of tyrosine, tryptophan and alanine transport in fibroblasts from ADHD children

Kinetic parameter	Amino acid	Children with ADHD (n = 14)	Controls (n = 13)	*p*-value
***V_max_***	**Tyrosine**	15.1 (11.5-25.4)	15.5 (7.8-21.5)	0.617
	**Tryptophan**	1.5 (1.1-3.0)	2.0 (1.3-4.7)	0.039*
	**Alanine**	42.8 (36.3-64.9)	32.0 (22.7-63.4)	0.031*
***K_m_***	**Tyrosine**	20.0 (6.3)	18.8 (5.5)	0.645
	**Tryptophan**	13.6 (4.7)	21.1 (8.2)	0.010**
	**Alanine**	125.7 (41.2)	100.7 (30.1)	0.086

The results are presented as median (range) for V_max _values and as mean (standard deviation) for K_m _values. ADHD, indicates Attention Deficit/Hyperactivity Disorder, **V_max_**, indicates maximal transport capacity (nmol/min/mg protein) and **K_m_**, indicates affinity of binding sites for a specific amino acid (μmol/l). *Statistically significant (p ≤ 0.05) **Statistically significant (p ≤ 0.01)

### Tyrosine

There were no significant differences between the ADHD group and controls in any of the kinetic parameters regarding the tyrosine transport (*V_max_*; p = 0.617, *K_m_*; p = 0.645). The median *V_max_*for tyrosine transport was 15.1 (11.5-25.4) nmol/min/mg protein in the ADHD group and 15.5 (7.8-21.5) nmol/min/mg protein in the comparison group. The mean *K_m_*for tyrosine transport was 20.0 (6.3) μmol/L in the ADHD group and 18.8 (5.5) μmol/L in the comparison group.

### Tryptophan

The ADHD group had significantly decreased *V_max _*(p = 0.039) and *K_m_*(p = 0.010) for tryptophan transport in comparison to controls. The median *V_max_*for tryptophan transport in the ADHD group was 1.5 (1.1-3.0) nmol/min/mg protein and for the comparison group 2.0 (1.3-4.7) nmol/min/mg protein. The mean *K_m_*for tryptophan transport in the ADHD group was 13.6 (4.7) μmol/L and for the comparison group 21.1 (8.2) μmol/L.

### Alanine

They ADHD group had a significantly higher *V_max_*for alanine transport (p = 0.031) than the controls, but the *Km *for alanine transport did not differ significantly (p = 0.086). The median *V_max_*for alanine was 42.8 (36.3-64.9) nmol/min/mg protein in the group of children with ADHD, while the median *V_max_*for the comparison group was 32.0 (22.7-63.4) nmol/min/mg protein. The mean *K_m_*of alanine transport in the ADHD group was 125.7 (41.2) μmol/L and for the comparison group 100.7 (30.1) μmol/L.

## Discussion

The main finding in the present study was that the group with ADHD had a decreased *V_max_*of tryptophan transport and an elevated *V_max_*of alanine transport across the fibroblast cell membranes. A low *V_max _*implies that the transport systems have lower capacity for amino acid uptake, while an increased *V_max_*indicates the opposite. Hence, the children in the ADHD group had a decreased transport of tryptophan and an elevated transport of alanine through the cell membrane of fibroblasts. Several mechanisms could contribute to the differences found between the ADHD group and the controls; 1) altered expression of transporter proteins and/or mutation/s in the genes coding for the involved transporter proteins; 2) general changes in the cell membrane, such as a disturbed membrane phospholipid composition (MPC) that could be altering the structure of the transporter proteins embedded in the membrane, which in turn might change the functionality of the transporters; 3) the altered amino acid transport could be caused by some other molecule(s) affecting the transporter(s) indirectly, e.g. some cytokines are known to influence amino acid uptake [35-39].

The ADHD group also had a decreased *K_m _*for tryptophan transport, which corresponds to an increased affinity between the amino acid and the transport protein, indicating that a lower concentration of extracellular tryptophan is needed to reach maximal transport capacity. This could be a compensatory mechanism to the decreased *V_max_*. However, there was a negative correlation between the *V_max_*and *K_m_*values for tryptophan transport within the ADHD group (r = -0.70, p = 0.005).

The use of fibroblast cells as a human experimental model to study amino acid transport across the BBB has gained support by a number of previous studies [15,23-29]. Moreover, the amino acid transport systems (i.e. system L and A) and their isoforms are expressed in both fibroblasts and at BBB [15,40]. The present findings of altered amino acid transport in fibroblasts from children with ADHD might therefore also be present at the BBB.

### Implications of an altered tryptophan transport

The decreased transport of tryptophan that was found in the ADHD group in the present study may imply reduced levels of serotonin in the CNS that might result in a disturbed serotonergic neurotransmission. A dysfunctional serotonergic system might secondarily lead to disturbances in the catecholaminergic systems as these neurotransmitter systems have strong anatomical and functional interactions [41].

Although the neurotransmitter most clearly implicated in ADHD is dopamine, there is a considerable amount of literature associating the serotonergic system with impulsivity, i.e. a core symptom in the combined type of ADHD [42]. There are also evidences suggesting that serotonin may be important in the action of amphetamine to reduce impulsive behaviour, potentially via its interactions with the dopaminergic system [43]. The dopamine-serotonin interaction and evidences for an altered dopaminergic and serotonergic contribution to ADHD were reviewed comprehensively by Oades [9]. The author presented studies supporting the role of serotonergic activity in impulse responses and implications as potential target for pharmacotherapy. Moreover, adolescents with ADHD and disruptive behaviour were studied by Malmberg et al (2008) with respect to MAO-A and 5-HTT genes and platelet MAO-B activity. The importance of further investigations of the serotonergic system, in addition to the dopaminergic system, in individuals with ADHD and disruptive behaviour disorders was emphasized [44].

If the transport of tryptophan is decreased through the BBB, as our results indicate, increasing plasma concentration of tryptophan (e.g. via tryptophan supplementation) might not result in more serotonin production. This could be one reason for the inconsistent results for tryptophan supplementation approaches in the treatment of ADHD [45]. However, some children with ADHD not responding to single treatment of psychostimulants are given a combination with selective serotonin reuptake inhibitors (SSRI) and show beneficial response.

### Implications of an altered alanine transport

The physiological relevance of the increased alanine transport that was found in the ADHD group has not been explored. However, at physiological plasma concentrations there is a competition between amino acids for transport across the BBB [13-15]. Although alanine is not involved in the synthesis of neurotransmitters, an elevated transport of alanine might influence the transport of other amino acids that are of essential importance for normal brain activity. Moreover, since alanine is involved in many complex and important metabolic pathways [46,47], our experiments cannot exclude that the elevated alanine transport might be related to other metabolic mechanisms of vital importance for normal brain activity. An increased transport of alanine has also been found in children with autism. ADHD and autism have high co-morbidity [48,49], and the present finding might imply a shared amino acid transport disturbance.

### Implications of an unaltered tyrosine transport

We did not find any significant differences in the transport of tyrosine between the ADHD group and the control group. Since tyrosine and tryptophan are considered to be transported across the BBB in similar fashion (i.e. through the LAT1 isoform of system L) it is difficult to explain why the tyrosine transport was not altered, whilst the tryptophan transport was. However, this indicates that it is not the overall capacity (expression) of system L that is affected; rather the isolated decrease in the *V_max_*for tryptophan could be linked to a more general alteration in plasma membrane function in ADHD. Altered membrane composition has been implicated in other psychiatric disorders such as schizophrenia and bipolar disorder [50-53]. Moreover, we measured the total transport of respective amino acids, i.e. we did not differentiate between the different transport systems and their isoforms.

### Possible transporters involved in the altered amino acid transport found in the ADHD group

In fibroblasts the tryptophan transport is through different amino acid transport systems at different substrate concentrations [22]. In this study we measured the transport of tryptophan at low concentrations (5 μM-500 μM of tryptophan), i.e. at physiological plasma concentrations of tryptophan (approximately 50 μM, physiological plasma levels of tyrosine and alanine are approximately 84 and 250 μM respectively). At this concentration, tryptophan is being transported through the LAT1 isoform of system L (approximately 80%) and through a high affinity system, which could be a hitherto undefined transporter or a variant of a known transport system with different functional properties due to an altered structure or conformation of the transporter protein. For example, system L is known to alter the transport properties due to different light-chain subunits [54,55]. The present results could thus imply that it might be the undefined tryptophan transporter that is malfunctioning in ADHD and not the LAT1 transporter, since the tyrosine transport was not altered. If this undefined transporter of tryptophan also exists at the BBB is not known to date. However, tyrosine is also transported through the ATA2 isoform of system A, which is the major transporter of alanine. The alanine transport was increased in the ADHD group, indicated by an increased *V_max_*but unchanged *K_m_*, and this transport disturbance could be caused by different mechanisms (see above), such as a higher expression of the ATA2 transporter protein in the fibroblasts from children with ADHD. Hence, if the LAT1 transporter is disturbed, tyrosine could compulsorily be transported through the ATA2 transporter and therefore the *V_max_*of tyrosine transport will not be affected in the ADHD group.

### Limitations

There are certain limitations with the present study. The patient group was relatively small, i.e. fourteen children with ADHD. The plasma levels of amino acids were not determined at the time of the biopsy collection. A further limitation is that only boys were included. However, our intention was to include children with a very similar phenotype of ADHD according to gender, age, type of ADHD and etiology. To achieve this we selected only boys (with the combined type of ADHD) in a rather narrow age span and for almost all boys there were close relatives/family members with the same disorder. Moreover, the patients were on different medications at the time of the biopsy collection, but it seems rather unlikely that this would affect the results as the cultivated fibroblasts were seeded for multiple generations *in vitro *before being used in the experiments [16].

## Conclusions

In conclusion, children with ADHD may have a decreased access of tryptophan and an elevated access of alanine in the brain. The decreased tryptophan availability in the brain might cause disturbances in the serotonergic neurotransmitter system, which secondarily might lead to changes in the catecholaminergic system. The physiological relevance of an increased access of alanine in the CNS has not been explored. However, this study was made *in vitro*, which makes it rather difficult to translate the results into *in vivo *situations. A further and extended exploration concerning the disturbance of amino acid transport in children with ADHD is thus necessary. Such exploration should include molecular investigations, looking for polymorphism in gene loci, further transport studies including girls with ADHD, measure the amount of serotonergic receptors in fibroblasts and study the effects of different molecules, such as cytokines, on amino acid uptake.

## Competing interests

The authors declare that they have no competing interests.

## Authors' contributions

**JJ**, carried out 70% of primary cultures from skin biopsy specimens, all tryptophan and alanine transport experiments, literature searches, statistical analysis and wrote the first draft of the manuscript. **ML**, participated in the design of the study and in the recruitment of the patients. **EF**, participated in the design, coordinated the recruitment of the patients and controls and contributed to the manuscript writing. **RV**, carried out 30% of primary cultures from skin biopsy specimens, and all tyrosine transport studies. **AÅ**, participated in the design of the study. **LB**, participated in the design of the study. **NV**, conceived, participated in the design, took the biopsies, interpreted the findings and coordinated the whole study. All authors have contributed to and approved the final manuscript.
